# Huge magnetoresistance in topological insulator spin-valves at room temperature

**DOI:** 10.1038/s41598-021-91242-y

**Published:** 2021-06-03

**Authors:** Peng Tseng, Jyun-Wei Chen, Wen-Jeng Hsueh

**Affiliations:** Nanomagnetism Group, Department of Engineering Science and Ocean Engineering, National Taiwan University, 1, Sec. 4, Roosevelt Road, Taipei, 10617 Taiwan

**Keywords:** Magnetic properties and materials, Spintronics, Topological matter

## Abstract

Topological insulators (TI) have extremely high potential in spintronic applications. Here, a topological insulators thin-film (TITF) spin valve with the use of the segment gate-controlled potential exhibits a huge magnetoresistance (MR) value higher than 1000% at room temperature which is more than 50 times the MR of typical topological insulators (TI) spin-valves. A high spin-polarized current is provided by the band structure generated by the tunable segment potential. The results reveal a very large resistance difference between the parallel and antiparallel configurations. The MR effect is strongly influenced by the thin-film thickness, the gate potential, the gate size, and the distribution. The proposed results will help to not only improve the room-temperature performance of the spin-valves but also enhance the applications of magnetic memories and spintronic devices.

## Introduction

Spintronics, an emerging mechanism in physics, has tremendous potentials for the development of memory storages such as magnetoresistive random access memories (MRAMs) to replace the traditional memory technologies^1–3^. Magnetoresistance (MR) effect provides an important target for spin operation in ferromagnet/barrier/ferromagnet magnetic tunnel junctions (MTJs). High MR ratio exceeding 400% is obtained by applying a crystalline magnesium oxide (MgO)^3–5^ and different barrier structures^6–8^ . In recent studies, a novel material, graphene, has attracted considerable attention as a two-dimensional Dirac material in spintronics^9,10^. Another famous Dirac material, topological insulator (TI), which has known for its insulator state with a bulk band gap and gapless surface states of linear dispersion, has been discovered to have good transport properties because of the protection of the time-reversal symmetry^11–16^. The latest discovery of graphene and TI heterostructures revealed considerable features in spintronic because of their exceptional spin properties^17,18^. Recently, the excellent spin properties are a great advantage in a TI and a ferromagnetic insulator (FI) junction^19–23^. These properties of TI-based devices reveal high reliability for spin applications to overcome the obstacles of quantum tunneling in conventional semiconductor junctions.

Researchers have explored many concepts to investigate novel spin properties in TI-based devices^24–26^. Based on the theoretical calculation of the non-equilibrium spin-polarized current, Taguchi et al*.* reported an investigation into a two-ferromagnet junction on the surface of typical TI. The results exhibited the MR value up to 29% and 67% in the research^25^. The structure of the two-dimensional ferromagnet/gate/ferromagnet junction on the surface of a typical TI showed a theoretical MR at zero temperature approach to 1000%^26^. The study reported that the alternate spin conductance is the main reason for the oscillating MR at zero temperature. Recently, an experimental report has shown a measured MR of 8% with a potential WS_2_ spin valve at low temperature^27^. Since most studies have focused on the MR at low temperature, the investigations are very difficult to apply in practice owing to the considerable number of limitations. Recently, a gap opened at the Dirac point rather than gapless (semimetallic) surface state has been observed in TI thin-films (TITFs)^22^. This extraordinary property reveals a high-potential advantage for spin application in Dirac materials. As far as we know, the analysis of the current feature and the MR effect in TITF spin-valves at room temperature is rare.

A TITF spin-valve with a segment gate-controlled potential is proposed to enhance the MR value. In this study, a thin film of TI such as BiSbTeSe_2_ is used in the spin valve instead of the typical bulk TI. The results show a high room-temperature MR value of more than 1000% adopting the band structure. The current is strongly restricted in the antiparallel configuration, but the parallel current reveals a high transmission probability of the spin electrons. The feature exhibits significant tunable behavior of the spin transport to novel spin-valve devices. The proposed method is qualitatively and quantitatively demonstrated with the research articles^25,26^.

## Model and methods

**Figure 1:**
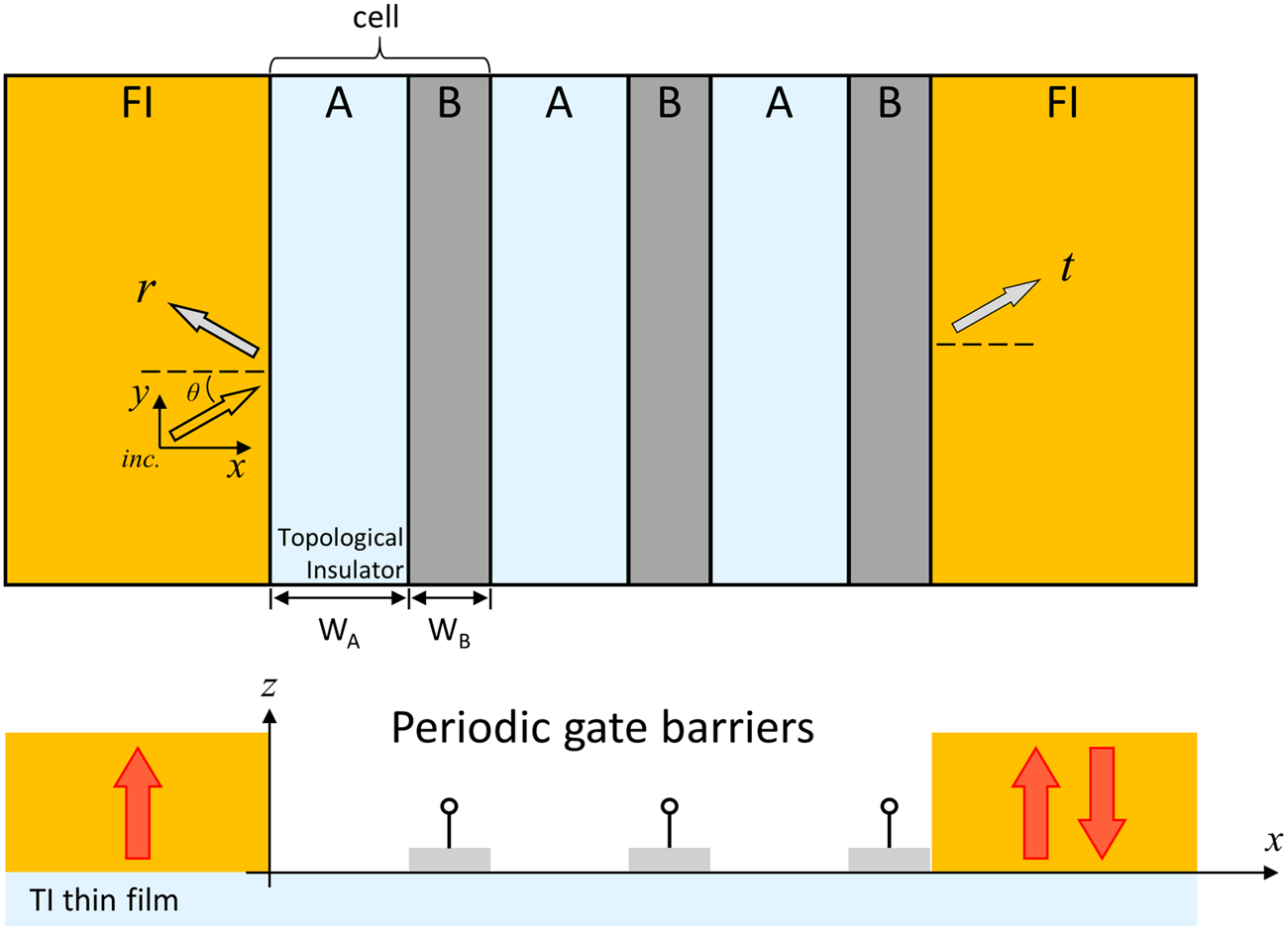
Schematic representation and device profile of a lateral TITF spin valve with a 3-cell top gate. The normal and top-gate potentials are taken as the unit cell (A/B) in this system. The top gates on the TI surface represented by the gray electrodes induce squared gate voltages *V*_*g*_. Spin electrons are injected into the spin valve with an incident angle *θ*.

Initially, two suitable FI electrodes, such as EuO and EuS, separated by the electrostatic-gate and normal surface deposited on a TITF material are considered, as shown in Fig. 1. The directions of the red arrows represent the magnetization by FI strips. The orange FI strips apply the magnetization on the surface of the TITF by the magnetic proximity effect. The orientations of the ferromagnet are parallel and antiparallel with the $- \infty$ and ${ + }\infty$ lengths taken to be semi-infinite^28^. The middle segment gate-potential along the *x*-axis consists of top-gate regions B and non-gate regions A, with the lengths *w*_*A*_ and *w*_*B*_. In our scheme, top gates applied to the TITF junction with oxide spacer are utilized to alter carrier concentration only within the gate region. Moreover, experimental research reported another alternative using a 3D p-type TI as a tunable gate which arouses a strong spin behavior such as spin-galvanic effect in the device^17^. The magnetization usually along the *z*-axis induces exchange splitting at the Dirac cones. The phenomenon between FI/graphene or FI/TI heterostructures with proximity exchange splitting has been showed in some studies^23,29,30^.

On the basis of the description of the two-dimensional Dirac fermions, the Hamiltonian that acts on the spinor of the TI surface state^30^ is given by

1 $$H = v_{F} \mathop{\sigma }\limits^{\rightharpoonup} \cdot \mathop{p}\limits^{\rightharpoonup} + \Delta_{s} \sigma_{\mu } + V(x) - \xi \Delta_{p} ,$$

where $\mathop{\sigma }\limits^{\rightharpoonup} = \left( {\sigma_{x} ,\sigma_{y} ,0} \right)$ is the Pauli matrix, $\mathop{p}\limits^{\rightharpoonup} = \left( {\hbar k_{x} ,\hbar k_{y} ,0} \right)$ denotes the in-plane momentum operator, *V*(*x*) is the finite top-gate potential, $\Delta_{s}$ denotes the Dirac gap opened by using magnetic doping or intersurface hybridization on TITF^19,22,31^, $\Delta_{p}$ is the proximity-induced exchange splitting on the TITF surface state with the spin-up ($\xi = + 1$) and spin-down ($\xi = - 1$) electrons^20^, and $v_{F}$ is the Fermi velocity.

The dispersion is equal to

2 $$E = eV_{j} - \xi \Delta_{p} + \left[ {\left( {\hbar v_{F} k_{x,j}^{\xi } } \right)^{{2}} + \left( {\hbar v_{F} k_{y}^{\xi } } \right)^{{2}} + \Delta_{s}^{2} } \right]^{{{1 \mathord{\left/ {\vphantom {1 2}} \right. \kern-\nulldelimiterspace} 2}}}$$

when charge carriers polarized in the eigenspinor orientations are injected into the system. While the range of the incident angle is $- {\pi \mathord{\left/ {\vphantom {\pi 2}} \right. \kern-\nulldelimiterspace} 2} \le \theta \le {\pi \mathord{\left/ {\vphantom {\pi 2}} \right. \kern-\nulldelimiterspace} 2}$, the conserved lateral and transverse wave vectors of the *y* and *x* directions to the boundary in the each region can be expressed as $k_{y}^{\xi } (E,\theta ) = {{\left[ {\left( {E - eV_{j} + \xi \Delta_{p} } \right)^{2} - \Delta_{s}^{2} } \right]^{{{1 \mathord{\left/ {\vphantom {1 2}} \right. \kern-\nulldelimiterspace} 2}}} \sin \theta } \mathord{\left/ {\vphantom {{\left[ {\left( {E - eV_{j} + \xi \Delta_{p} } \right)^{2} - \Delta_{s}^{2} } \right]^{{{1 \mathord{\left/ {\vphantom {1 2}} \right. \kern-\nulldelimiterspace} 2}}} \sin \theta } {\hbar v_{F} }}} \right. \kern-\nulldelimiterspace} {\hbar v_{F} }}$ and $k_{x,j}^{\xi } (E,\theta ) = {\text{sgn}} (\eta_{j}^{\xi } ){{\sqrt {\left[ {\left( {E - eV_{j} + \xi \Delta_{p} } \right)^{2} - \Delta_{s}^{2} } \right] - \left( {k_{y}^{\xi } } \right)^{2} } } \mathord{\left/ {\vphantom {{\sqrt {\left[ {\left( {E - eV_{j} + \xi \Delta_{p} } \right)^{2} - \Delta_{s}^{2} } \right] - \left( {k_{y}^{\xi } } \right)^{2} } } {\hbar v_{F} }}} \right. \kern-\nulldelimiterspace} {\hbar v_{F} }}$, respectively. By meaning of the eigenstates, $$\Psi_{j} = \left[ {\begin{array}{*{20}c} {\Phi_{j}^{ + } } & {\Phi_{j}^{ - } } \\ \end{array} } \right]^{T}$$, the envelope functions in the segment potential and the FI region can be written as two spinors, $$\Phi_{j}^{\xi } = \frac{{e^{{ik_{y} y}} }}{\sqrt 2 }\left( {\begin{array}{*{20}c} {e^{{ik_{x,j} x}} } & {e^{{ - ik_{x,j} x}} } \\ {e^{{i\left( {\phi_{j} + k_{x,j} x} \right)}} } & { - e^{{ - i\left( {\phi_{j} + k_{x,j} x} \right)}} } \\ \end{array} } \right)\left( {\begin{array}{*{20}c} {a_{j}^{\xi } } \\ {b_{j}^{\xi } } \\ \end{array} } \right)$$ in each *j* segment. Here, $a_{j}^{\xi }$ and $b_{j}^{\xi }$ are the wave amplitudes of incidence and reflection, respectively, and $\phi_{j}$ is the relationship of $\phi_{j} = {\text{arccos}}\left\{ {{{k_{x,j} } \mathord{\left/ {\vphantom {{k_{x,j} } {\left[ {\left( {E - eV_{j} + \xi \Delta_{p} } \right)^{2} - \Delta_{s}^{2} } \right]}}} \right. \kern-\nulldelimiterspace} {\left[ {\left( {E - eV_{j} + \xi \Delta_{p} } \right)^{2} - \Delta_{s}^{2} } \right]}}} \right\}$. On the basis of the ballistic transport in the junction, inelastic scattering is ignored, and the spin-polarized current in the *x*-direction can be expressed by the Landauer formulation^32^

3 $$I_{SD}^{\xi } = I_{0} \int_{ - \infty }^{\infty } {\int_{{ - \frac{\pi }{2}}}^{{\frac{\pi }{2}}} {T^{\xi } (E,\theta ,V_{SD} )} } \left[ {f_{S} \left( {E - \varepsilon_{S} } \right) - f_{D} \left( {E - \varepsilon_{D} } \right)} \right]\cos \theta d\theta \left| E \right|dE,$$

where $I_{0} = {{eW_{y} } \mathord{\left/ {\vphantom {{eW_{y} } {h^{2} }}} \right. \kern-\nulldelimiterspace} {h^{2} }}v_{F}$, the functions of $f_{S} = \left\{ {\exp \left[ {\left( {E - \varepsilon_{S} } \right)/k_{B} T} \right] + 1} \right\}^{ - 1}$ and $f_{D} = \left\{ {\exp \left[ {\left( {E - \varepsilon_{D} } \right)/k_{B} T} \right] + 1} \right\}^{ - 1}$ are the Fermi–Dirac distribution functions of the source (S) and the drain (D) electrodes, respectively; $\varepsilon_{S}$ and $\varepsilon_{D}$ denote the Fermi levels on the S and the D, respectively; and $T^{\xi } (E,\theta ,V_{SD} )$ is the bias-dependent transmission probability of the entire system. *W*_*y*_ denotes the width of the TITF, and the Boltzmann constant *k*_*B*_ is 1.38 × 10^−23^ J/K. The integral form of Eq. (3) has been coded in MATLAB to obtain current features. The MR ratio of this device is defined as $MR(\% ) = {{\left( {I_{P} - I_{AP} } \right)} \mathord{\left/ {\vphantom {{\left( {I_{P} - I_{AP} } \right)} {I_{AP} }}} \right. \kern-\nulldelimiterspace} {I_{AP} }} \times 100\%$ with the current in parallel and antiparallel configurations.

## Results and discussion

**Figure 2:**
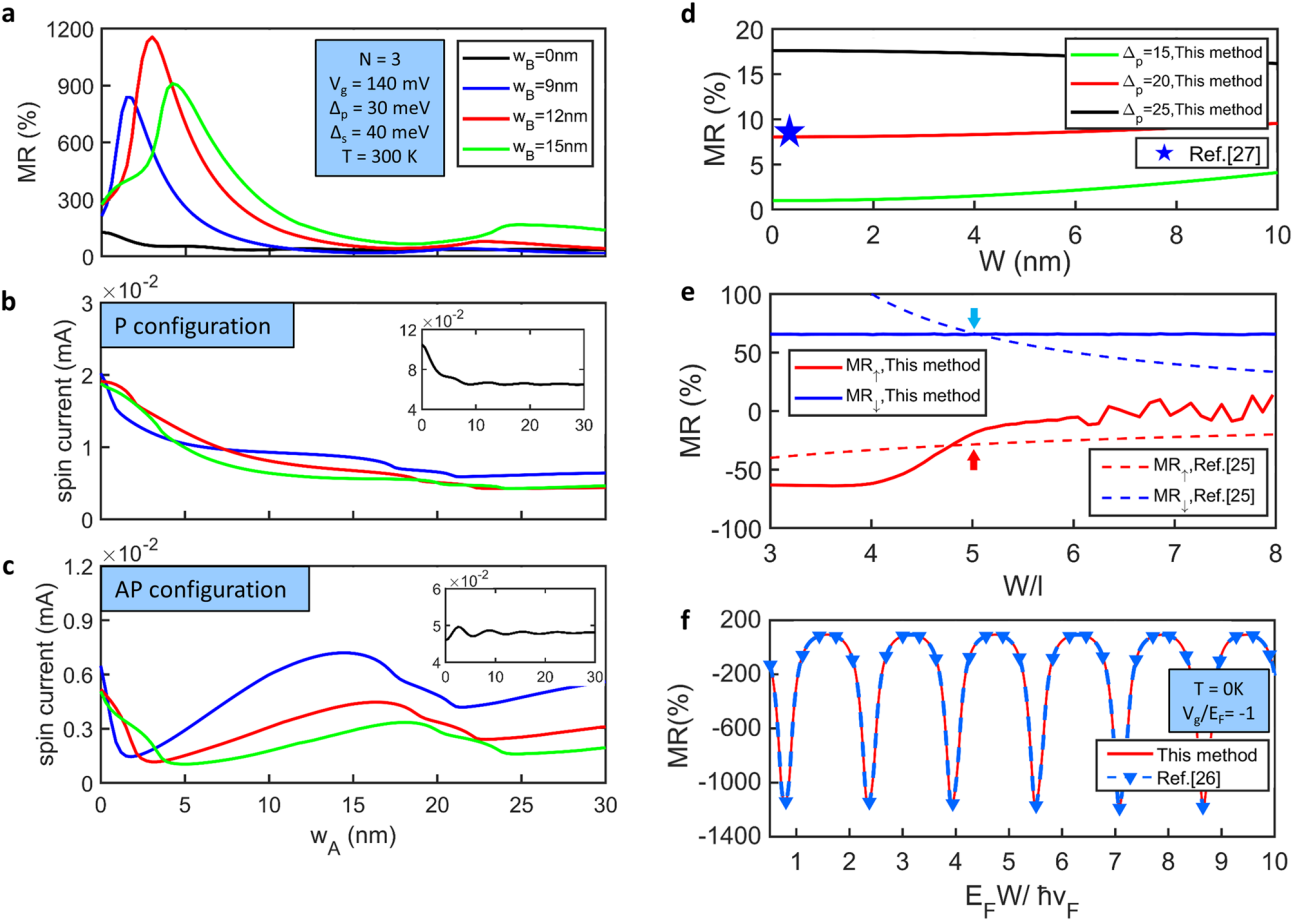
(**a**) Room-temperature MR ratios versus the length between two gates in 3-cell spin valves with *w*_*B*_ = 0 nm, *w*_*B*_ = 9 nm, *w*_*B*_ = 12 nm, and *w*_*B*_ = 15 nm. Spin-polarized current in the (**b**) parallel and (**c**) antiparallel configurations as a function of *w*_*A*_ with different gate lengths at room temperature. The insets depict the spin-polarized currents in a standard TITF spin valve (*w*_*B*_ = 0 nm). (**d**) MR ratio obtained by the proposed TITF spin valve with different proximity exchange splitting at an extremely low temperature, T = 4 K. (**e**)(**f**) MR simulation corresponding to the works described in references^25,26^ 25, 26, theoretically. *l* and *E*_*F*_ are the mean-free path and the Fermi energy.

Performance of the MR variation and the spin-polarized current for different lengths of the top-gate potential is a very important target in spin valves. The detailed MR curves for the different lengths between two gates in a 3-cell segment potential TITF spin-valve are shown in Fig. 2a. The system parameters are as follows: gate voltage *V*_*g*_ = 140 mV, gap at the Dirac cone Δ_*s*_ = 40 meV, exchange splitting Δ_*p*_ = 30 meV, period number N = 3, temperature T = 300 K, and Fermi energy *ε*_*F*_ = 40 meV. All analysis programs were conducted in the platform of MATLAB 2019a for running the simulation. The MR peaks appear at a small distance between two gates, *w*_*A*_ < 5 nm, where the maximum MR ratio shows a magnitude of more than 1150%, as indicated by the red curve in Fig. 2a. The MR value is still up to 900% when *w*_*B*_ = 12 nm. The difference in the MR is considerably small, while *w*_*A*_ exceeds 15 nm. Furthermore, the MR performance of a standard TITF spin-valve without the gate potential (*w*_*B*_ = 0 nm) is ordinary of about 53%, as indicated by the black curve in Fig. 2. Compared to the standard spin valve, the MR value increase almost 21 times by using segment-potential modulation. To determine the reason for the MR variation, the spin-polarized currents in the parallel and antiparallel configurations are analyzed. In general, the currents are severely affected by the distribution of the segment potentials. These current features show that there is an obvious drop in the antiparallel configuration, particularly, as shown in Fig. 2c. The spin-polarized current in the antiparallel mode can be approximately only one order of magnitude smaller than that under the normal condition. However, the three currents corresponding to the parallel magnetization alignment are almost in the similar magnitude for the variation of the segment potential, as plotted in Fig. 2b.

The difference between the currents of the parallel-mode and the antiparallel-mode is the main reason for the huge MR change. The current features are qualitatively and quantitatively demonstrated in a typical TI device, as described in the reference^33^. It is found that the current feature shown in Fig. 2 is very different from the monotonous increase obtained in the research^33^, which imply that the transport properties can be efficiently controlled in the proposed spin valve. Furthermore, it is found that the three antiparallel current has obvious growing when *w*_*A*_ > 9 nm. Compared to the traditional quantum systems, it is different from the system using a huge tunneling current in the parallel configuration with a very small resistance. In the present work, such a unique mechanism in this system is attributed to the obvious transport suppression of the spin electrons in the antiparallel configuration. The spin-polarized currents in the antiparallel configuration dominate the main contributor to the MR and the position of the maximum peak. These conditions are the most important to provide significant resistance changes in the different configurations of the spin valve.

Considering the fundamental model, the performance of the typical TI spin-valve (without the gate potential) with a linear dispersion relation (Δ_*s*_ = 0 meV), expressed as S/TI/D, is proposed when the exchange splittings have several variations. It is found that the MR value increases slightly as Δ_*p*_ goes up from 15 to 25 meV. The maximum MR value up to 10 times of that for Δ_*p*_ = 15 meV is observed. The length (thickness) of the TI is taken as *W*. Moreover, other validations of the theoretical studies show that the MR ratios for the spin-up and spin-down polarization in the reference^25^ are shown in Fig. 2e. The corresponding MR ratios are 22.56% and 65.18% with 24.1% and 5.8% errors for the case *W*/*l* = 5. In Fig. 2f, the results exhibit the conductance calculation at the absolute zero temperature is in high accordance with the work reported in reference^26^.

**Figure 3:**
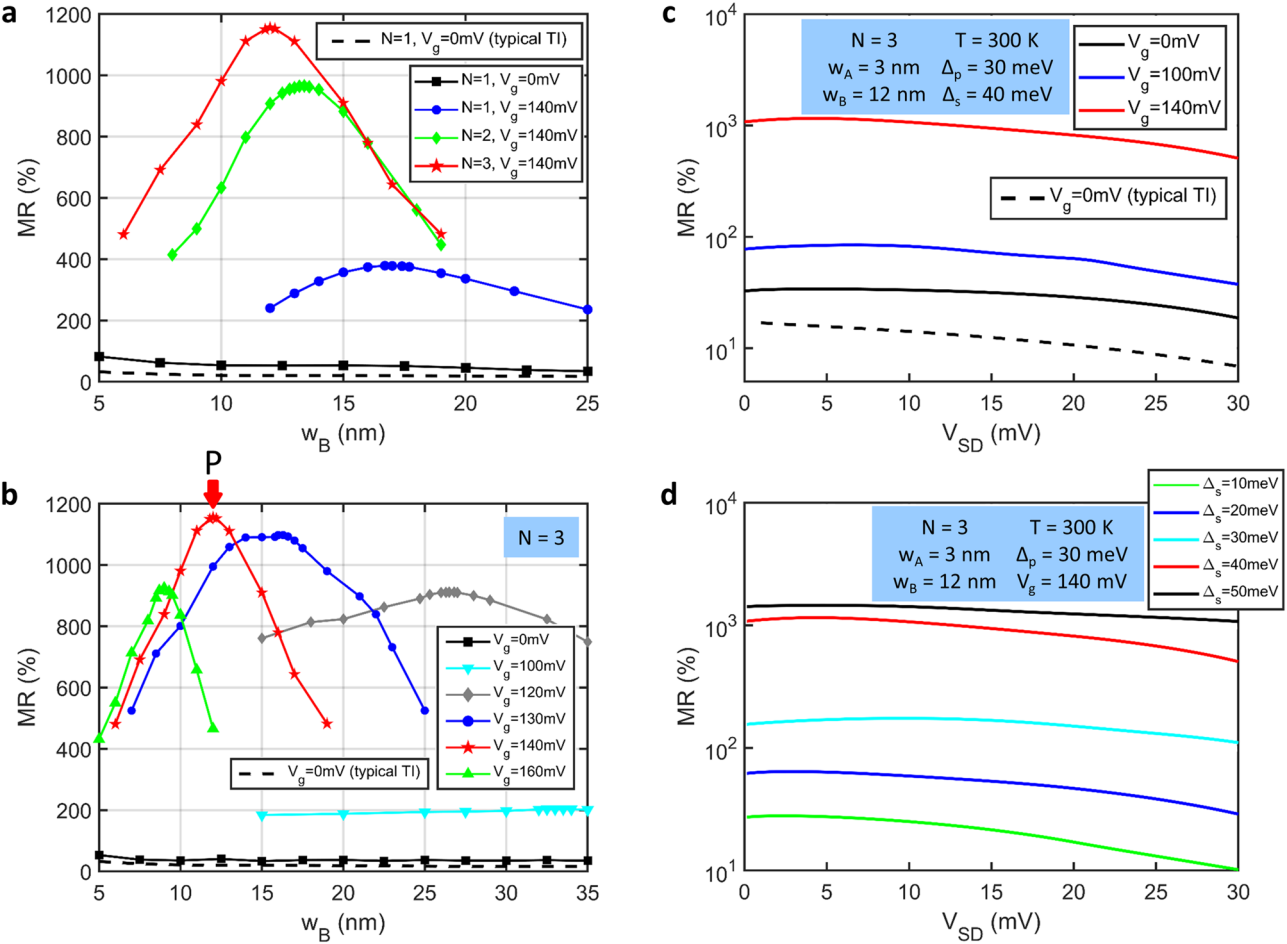
(**a**) Room-temperature MR ratio versus gate length in TITF spin valves with different period number N. The black and black-dashed curves represent the MR ratio of the standard TITF and typical TI spin valves without the gate potential, respectively, where the value of *w*_*B*_ is taken as the period length Λ_*p*_ (Λ_*p*_ = *w*_*A*_ + *w*_*B*_). (**b**) Room-temperature MR ratio versus gate length with different potential heights for N = 3. Here, *w*_*B*_ indicates the period length Λ_*p*_ when *V*_*g*_ = 0 mV. The corresponding value of the length *w*_*A*_ is given in Supplementary Information for details. The other parameters are the same as those shown in Fig. 2a. (**c**) Bias-dependent MR value for the 3-cell potential spin valve at room temperature with different *V*_*g*_. (**d**) Bias-dependent MR value with different Dirac gap Δ_*s*_.

To further understand the phenomena of the segment potential, the trend of MR variation for the different lengths and heights are shown in Fig. 3. In Fig. 3a, the MR ratio exceeds 950% when N = 2. The corresponding value of *w*_*A*_ is given in Supplementary for details. When the period number increases to three cells, the MR value shows an obvious peak higher than 1150%, as shown by the red curve in Fig. 3a. The migration path of the maximum MR peaks is shifting to the bottom-right corner of the chart in the presence of the N increase. That is, the devices require only a smaller gate length to obtain the better MR performance with a large period number. However, it is not worth manufacturing a multi-electrode segment potential in the devices to obtain limited improvements. Moreover, the MR value is lower than 100% in a standard TITF spin-valve, as illustrated by the black line in Fig. 3a. Under this condition, the *x*-axis of *w*_*B*_ is taken as the total TITF channel length Λ_*p*_ (Λ_*p*_ = *w*_*A*_ + *w*_*B*_). The giant MR is enhanced approximately 15 times higher than that in the standard TITF spin valve, where the MR value increase from 80 to 1150%. The MR value is below 30% in the typical TI spin-valve. These giant MR features are attributed to the mechanism of the band structure from the periodic potential^8^ that has a significant correlation with the spin-polarized current. The results show that the forbidden and allowed bands can modulate the electron transport of the parallel and antiparallel configurations, influenced by the appropriate design of the segment potential. The forbidden band limited the electron transmission in the antiparallel mode plays the role of the main contributor on the basis of the good conductor of the TI surface state, resulting in the large system resistance.

The potential height is also a factor that greatly influences the MR. When the potential height *V*_*g*_ is selected as 120 mV, about three times the Fermi level, the maximum MR peak is approximately 900%, as shown in Fig. 3b. It is observed that the MR graph has a smooth curve for the potential height of less than 120 mV. While the potential height exceeds a critical voltage, 150 mV, the MR peaks decrease to below 1000%. Moreover, for *V*_*g*_ > 140 mV, the MR curves decrease dramatically with a small gate change as compared to those under the low *V*_*g*_ condition. The maximum MR peak, point P, is situated at a gate length equal to 12 nm where the MR ratio is of up to 1160%, corresponding to the current features shown in Fig. 2b,c.

Then, the bias-dependent MR for different potential heights is shown in Fig. 3c. The length of the unit cell is fixed at *w*_*A*_ = 3 nm and that of *w*_*B*_ as 12 nm. The results show that the spin transport behavior sharply decreases when the potential height reduces from 140 mV. The MR ratios of the spin valve are decrease with an increase in the bias voltage. The MR ratio is still up to 600% even if a large bias is applied. The MR values are not more than 100% with *V*_*g*_ < 100 mV. For the standard case (*V*_*g*_ = 0 mV), the MR value (33%) is one-thirtieth lower than the maximum MR peak, as shown by the green curve in Fig. 3c. It is found that the segment potential causes a very important enhancement in the spin-valve device. The MR variation as a function of the bias with a different Dirac gap, Δ_*s*_, is shown in Fig. 3d. This figure clearly shows that the maximum MR ratio is up to approximately 1500% with the Dirac gap Δ_*s*_ = 50 meV. The MR value is enhanced by nearly 30% with a larger gap. Indeed, the larger intrinsic gap can provide a better transport restriction of spin electrons. When Δ_*s*_ is less than 30 meV, the MR value drops to below 180% in the 3-cell spin valve, corresponding to the aqua, blue, and green lines in Fig. 3d. In particular, the MR value significantly decreases to approximately 25% in the case of a small gap, Δ_*s*_ = 10 meV, as indicated by the green curve. Furthermore, the MR ratio for Δ_*s*_ = 50 meV shows a steadier smooth curve than the other graphs without an obvious reduction at the large bias.

**Figure 4:**
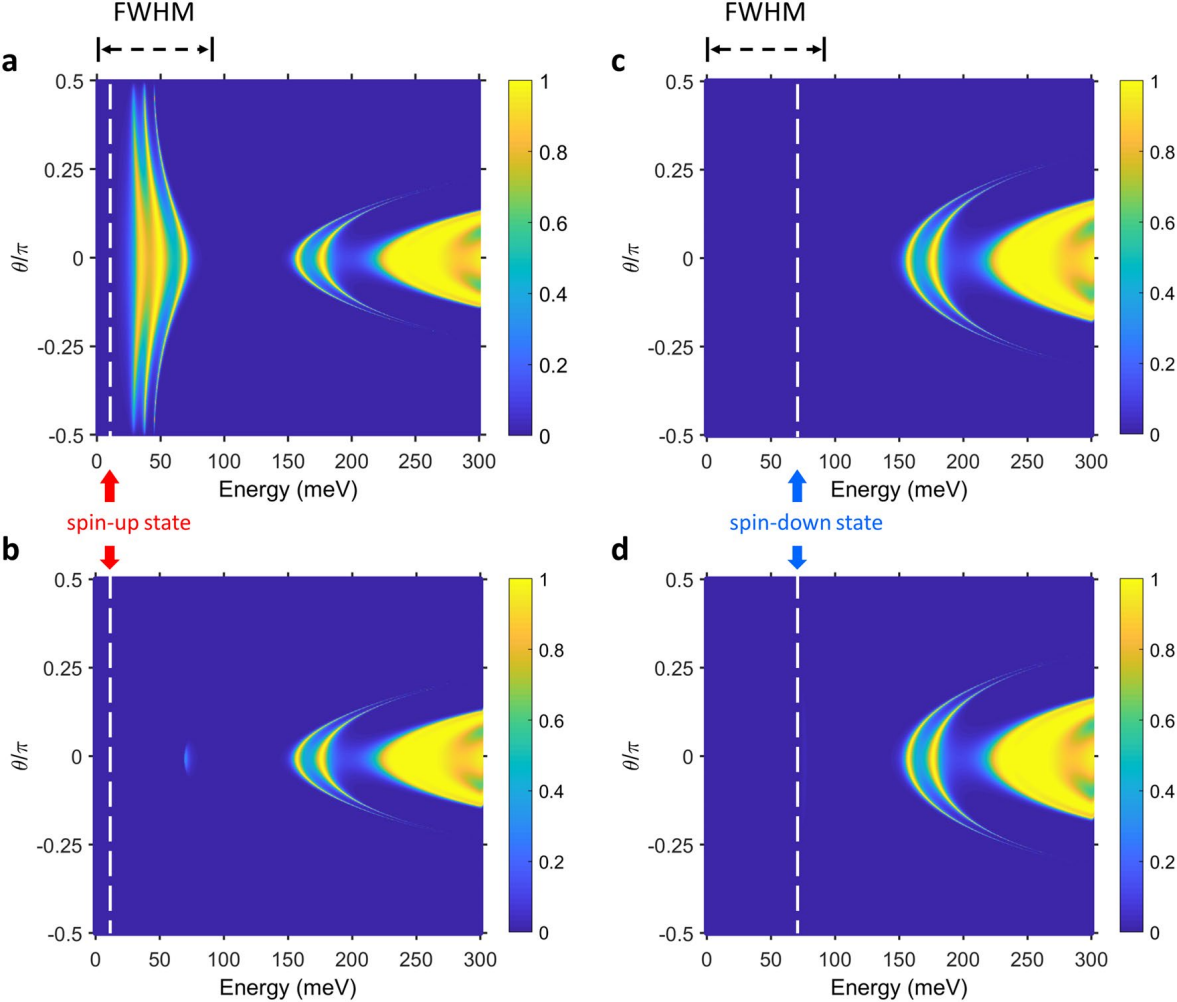
Transmission probabilities of each spin state for altering incident energy and incident angle at point P of Fig. 3b. The transmission probability of the spin-up electrons in the (**a**) parallel and (**b**) antiparallel configurations. (**c**,**d**) The transmission probability of the spin-down electrons in the parallel and the antiparallel. Here, the lengths of the gate and the non-gate regions are selected as *w*_*A*_ = 3 nm and *w*_*B*_ = 12 nm, respectively. The black-dashed lines above the figure indicate the full width at half-maximum (FWHM) of the Fermi–Dirac function. The white dashed lines and arrows indicate the origin of each spin polarization. The other parameters are the same as those considered in Fig. 2a.

Finally, Fig. 4 shows the influence of the transmission probability by varying the magnetization direction to analyze the variation of MR and current. Figures were produced by licensed MATLAB 2019a. The system parameters are selected as *w*_*A*_ = 3 nm and *w*_*B*_ = 12 nm, corresponding to point P in Fig. 3b. On the basis of the band structure theory, the eigenfunction of the band structure in the system can be obtained by solving the Bloch wave function, cos(*KD*), where *K* is the Bloch wave number, and *D* is the period length of *D* = *w*_*A*_ + *w*_*B*_. The results of the cos(*KD*) function reveal the ability of the spin-polarized electrons to propagate in the periodic potential. When the function has the solutions of |cos(*KD*)|< 1, the electrons can propagate through the system with the appropriate energy range. In contrast, the solutions of |cos(*KD*)|> 1 cause the attenuation of the electron transport. The results of the band structure can be described by the corresponding transmission probabilities in each spin state, $T^{\xi } (E,\theta ,V_{SD} )$, as shown in Fig. 4. Owing to the proximity-induced exchange splitting, the origin of the spin-up (spin-down) electrons are split into $\Delta_{s} - \Delta_{p}$ ($\Delta_{s} + \Delta_{p}$) with the Dirac gap in the TITF surface state. The transmission of the spin-up polarization has an individual allowed band within the half-maximum (FWHM) of the Fermi–Dirac function in the parallel configuration, as shown in Fig. 4a. However, the transmission probabilities under the other three circumstances are attributed to a lack of such transport mechanism. The transmission probabilities with energy under 150 meV sunk into the forbidden band for the spin-down electrons in the parallel configuration and the both spin electrons in the antiparallel configuration, as shown in Fig. 4b–d. These results are due to the combination of the spin splitting and the band modulation by the mismatch of the Fermi velocities in the segment potential. The spin valve with the periodic potential can turn off the low-angle transport channel, which is unable to achieve in the monotonic single-barrier and typical spin valves^26,33^.

According to Eq. (2), the superposition of the transmission probabilities and the Fermi–Dirac distribution will considerably determine the features of the spin-polarized currents. It is found that the gap (forbidden bands) is mostly located in the FWHM region producing the obvious current suppression in the antiparallel configuration, as shown in Fig. 4b,d. Similarly, Fig. 4c shows that the Fermi–Dirac function and the forbidden band are overlapping in the low-energy region for the spin-down electrons in the parallel configuration. In the high-energy region, the allowed bands of the transmission probabilities overlap with the tail of Fermi–Dirac distribution in both the configurations. The small currents are obtained. In contrast, the allowed band of the spin-up transmission probability in the parallel mode is located precisely in the FWHM region of the Fermi–Dirac function. The parallel current always maintains a steady transport with the alteration of the device. Thus, the transport of the parallel current remains strong and stable. On the basis of the transport properties in the parallel configuration, higher MR could be obtained by further restricting the current in the antiparallel configuration.

## Conclusions

In summary, the MR value of the TITF spin-valve exceeding 1000% at room temperature generated by the segment gate-controlled potential is achieved. The segment potential is an important factor to the MR effect. The giant MR effect is approximately 50 times larger than that in the typical TI spin-valve. The results show that the room-temperature MR value has significant variations upon an extreme suppression of the spin-polarized current in the antiparallel configuration as the potential height, the gate length, and the distribution change. The current phenomenon in the system is modulated by the superposition of the tunable band structure and the Fermi distribution. Moreover, the proposed method is demonstrated with the theoretical studies^25,26^. The research provides a potential development on advanced storage memories for spintronic devices.

## Supplementary Information

Supplementary Information.

## References

[1] Jiang J (2020). Concurrence of quantum anomalous Hall and topological Hall effects in magnetic topological insulator sandwich heterostructures. Nat. Mater..

[2] Friesen C (2019). Magneto-Seebeck tunneling on the atomic scale. Science.

[3] Wang M (2018). Current-induced magnetization switching in atom-thick tungsten engineered perpendicular magnetic tunnel junctions with large tunnel magnetoresistance. Nat. Commun..

[4] Parkin SSP (2004). Giant tunnelling magnetoresistance at room temperature with MgO (100) tunnel barriers. Nat. Mater..

[5] Ikeda S (2008). Tunnel magnetoresistance of 604% at 300K by suppression of Ta diffusion in CoFeB/MgO/CoFeB pseudo-spin-valves annealed at high temperature. Appl. Phys. Lett..

[6] Sharma A, Tulapurkar AA, Muralidharan B (2018). Band-pass Fabry-Pèrot magnetic tunnel junctions. Appl. Phys. Lett..

[7] Chen CH, Cheng YH, Ko CW, Hsueh WJ (2015). Enhanced spin-torque in double tunnel junctions using a nonmagnetic-metal spacer. Appl. Phys. Lett..

[8] Chen CH, Hsueh WJ (2014). Enhancement of tunnel magnetoresistance in magnetic tunnel junction by a superlattice barrier. Appl. Phys. Lett..

[9] Žutić I, Matos-Abiague A, Scharf B, Dery H, Belashchenko K (2019). Proximitized materials. Mater. Today.

[10] Tseng P, Hsueh WJ (2019). Ultra-giant magnetoresistance in graphene-based spin valves with gate-controlled potential barriers. New J. Phys..

[11] Bansil A, Lin H, Das T (2016). Colloquium: topological band theory. Rev. Mod. Phys..

[12] Rienks EDL (2019). Large magnetic gap at the Dirac point in Bi_2_Te_3_/MnBi_2_Te_4_ heterostructures. Nature.

[13] Zhang T (2019). Catalogue of topological electronic materials. Nature.

[14] Lin YC, Chou SH, Hsueh WJ (2020). Robust high-Q filter with complete transmission by conjugated topological photonic crystals. Sci. Rep..

[15] Wu H (2019). Room-temperature spin-orbit torque from topological surface states. Phys. Rev. Lett..

[16] Jost A (2017). Electron–hole asymmetry of the topological surface states in strained HgTe. Proc. Natl. Acad. Sci..

[17] Khokhriakov D, Hoque AM, Karpiak B, Dash SP (2020). Gate-tunable spin-galvanic effect in graphene-topological insulator van der Waals heterostructures at room temperature. Nat. Commun..

[18] Zhao B (2020). Unconventional charge–spin conversion in Weyl-semimetal WTe_2_. Adv. Mater..

[19] Chen YL (2010). Massive Dirac fermion on the surface of a magnetically doped topological insulator. Science.

[20] Yu R (2010). Quantized anomalous Hall effect in magnetic topological insulators. Science.

[21] Wray LA (2011). A topological insulator surface under strong Coulomb, magnetic and disorder perturbations. Nat. Phys..

[22] Xu Y, Jiang G, Miotkowski I, Biswas RR, Chen YP (2019). Tuning insulator-semimetal transitions in 3D topological insulator thin films by intersurface hybridization and in-plane magnetic fields. Phys. Rev. Lett..

[23] Haugen H, Huertas-Hernando D, Brataas A (2008). Spin transport in proximity-induced ferromagnetic graphene. Phys. Rev. B.

[24] Yang CY (2019). Direct observation of proximity-induced magnetism and spin reorientation in topological insulator on a ferrimagnetic oxide. Appl. Phys. Lett..

[25] Taguchi K, Yokoyama T, Tanaka Y (2014). Giant magnetoresistance in the junction of two ferromagnets on the surface of diffusive topological insulators. Phys. Rev. B.

[26] Zhang KH, Wang ZC, Zheng QR, Su G (2012). Gate-voltage controlled electronic transport through a ferromagnet/normal/ferromagnet junction on the surface of a topological insulator. Phys. Rev. B.

[27] Zatko V (2019). Band-structure spin-filtering in vertical spin valves based on chemical vapor deposited WS2. ACS Nano.

[28] Tseng P, Chen ZY, Hsueh WJ (2020). Superlattice-barrier magnetic tunnel junctions with half-metallic magnets. New J. Phys..

[29] Yang HX (2013). Proximity effects induced in graphene by magnetic insulators: First-principles calculations on spin filtering and exchange-splitting gaps. Phys. Rev. Lett..

[30] Yokoyama T, Tanaka Y, Nagaosa N (2010). Anomalous magnetoresistance of a two-dimensional ferromagnet/ferromagnet junction on the surface of a topological insulator. Phys. Rev. B.

[31] Ahn EC (2020). 2D materials for spintronic devices. NPJ 2D Mater. Appl..

[32] Blanter YM, Büttiker M (2000). Shot noise in mesoscopic conductors. Phys. Rep..

[33] Scharf B, Matos-Abiague A, Han JE, Hankiewicz EM, Žutić I (2016). Tunneling planar Hall effect in topological insulators: spin valves and amplifiers. Phys. Rev. Lett..

