# Supplementary material for: Huge magnetoresistance in topological insulator spin-valves at room temperature
Source: Sci Rep. 2021 Jun 3;11:11717. doi: 10.1038/s41598-021-91242-y (PMC8175690; doi:10.1038/s41598-021-91242-y)
Supplement: Supplementary file 1 — Supplementary Information. [file 41598_2021_91242_MOESM1_ESM.docx]

**Huge magnetoresistance in topological insulator spin-valves at room temperature**

**Peng Tseng, Jyun-Wei Chen, and Wen-Jeng Hsueh***

**SUPPLEMENTARY**

A 3-cell segment potential spin-valve on a topological insulator thin-film (TITF) is considered. The red arrows represent the magnetization direction of the FI strips. The spin splitting on the TITF is induced by the orange FI strip. The middle segment potential along the *x* axis comprises the top-gate region B and non-gate region A with the distances, *w_A_* and *w_B_*. The gate-controlled potential applied on the TI surface is *V_g_*.

Figure S1b shows the MR ratio at room temperature with 12 nm gate length. The parameters in the system are *V_g_* = 140 mV, Δ*_s_* = 40 meV, Δ*_p_* = 30 meV, N = 3, and $\varepsilon_{F}=40\text{ meV}$. The green arrow indicates the MR value with the parameter *w_A_* = 5.4 nm. The brown arrow indicates the MR value with *w_A_* = 12 nm. The MR ratio of Fig. S1b is corresponding to the red line in Fig. 2a of the main article. In figure S1b, it is clearly seen the MR peak at the small length with *w_A_* = 3 nm. The maximum MR ratio shows the magnitude exceeding 1150% in figure S1b. In the main text, the mechanism of the huge MR effect is discussed in depth by the spin-polarized and the transmission probabilities. Here, we investigate the case for the different MR conditions and its mechanism, as indicated by the arrows in figure S1b.

The transmission probabilities of the 3-cell segment potential spin-valve are shown in the Fig. S2. The length between two gates *w_A_* is selected at 5.4 nm. Compared to figure 4, the spin-up transmission probability is reduction within the energy window $\text{[}\Delta_{s}-\Delta_{p}\text{, }\Delta_{s}+\Delta_{p}\text{]}$ in the parallel configuration. The results lead to the large rise of the resistance in the parallel configuration. The lower MR phenomenon can be attributed to the significant decrease of the MR difference in the parallel and the antiparallel configurations. For the other three states, the transmission doesn't suffer much influence with *w_A_* change. The same location of the spin transport channels are presented corresponding to Fig. 4. Thus, the MR ratio is reduced by half of the maximum MR peak based on the decrease of the parallel-mode transmission, as illustrated by the green arrow in figure S1b.

Further, when the system continuously increases the length of *w_A_* the transmission probabilities of the system with fixed *w_A_* = 12 nm are shown in Fig. S3. It is clearly found that the transmission probability within the energy window $\text{[}\Delta_{s}-\Delta_{p}\text{, }\Delta_{s}+\Delta_{p}\text{]}$ dramatically reduced to the low level in the parallel configuration with regard to the circumstances in Figs. 4 and S2. The reduced transmission probability is located in FWHM to produce a relatively low spin-polarized current. The parallel-mode resistance is relatively higher than that in the conditions of Figs. 4 and S2. Thus, the parallel-mode domination is non-existent compared to the previous cases. Moreover, from figures S3b and S3d, it is seen that the spin channels of transmission probabilities are significant increase within the entire Fermi–Dirac distribution. There is a narrow spin channel (allowed band) appear at the incident energy near 100 mV. This mechanism causes a resistance decrease in the antiparallel configuration. Needless to say, the MR value is very ordinary (below 100%) while the device setup with a larger non-gate segment in the TI-based spin valve discussed above.


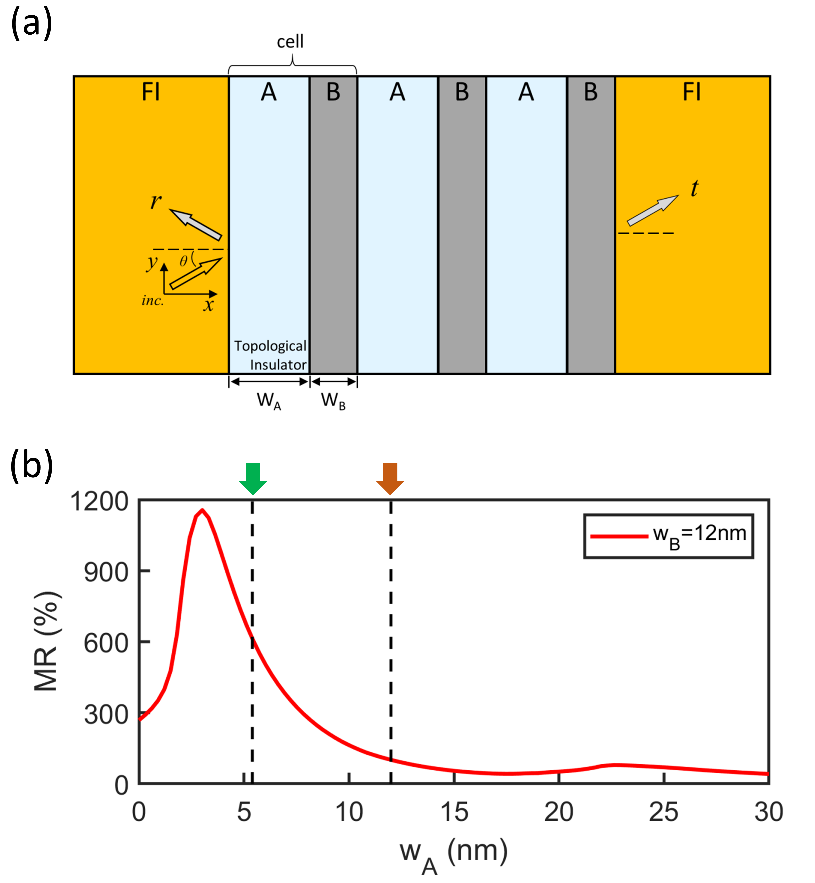


**Fig. S1** (**a**) Sketch diagram of a 3-cell potential spin-valve on the surface of the TITF as a lateral transport channel. The non-gate and top-gate segments are taken as the unit cell (A/B) in this structure. (**b**) Room-temperature MR ratio versus the length of non-gate region in 3-cell spin-valves with *w_B_* = 12 nm. The parameters are *w_B_* = 12 nm, *V_g_* = 140 mV, Δ*_s_* = 40 meV, Δ*_p_* = 30 meV, N = 3, and $\varepsilon_{F}=40\text{ meV}$.


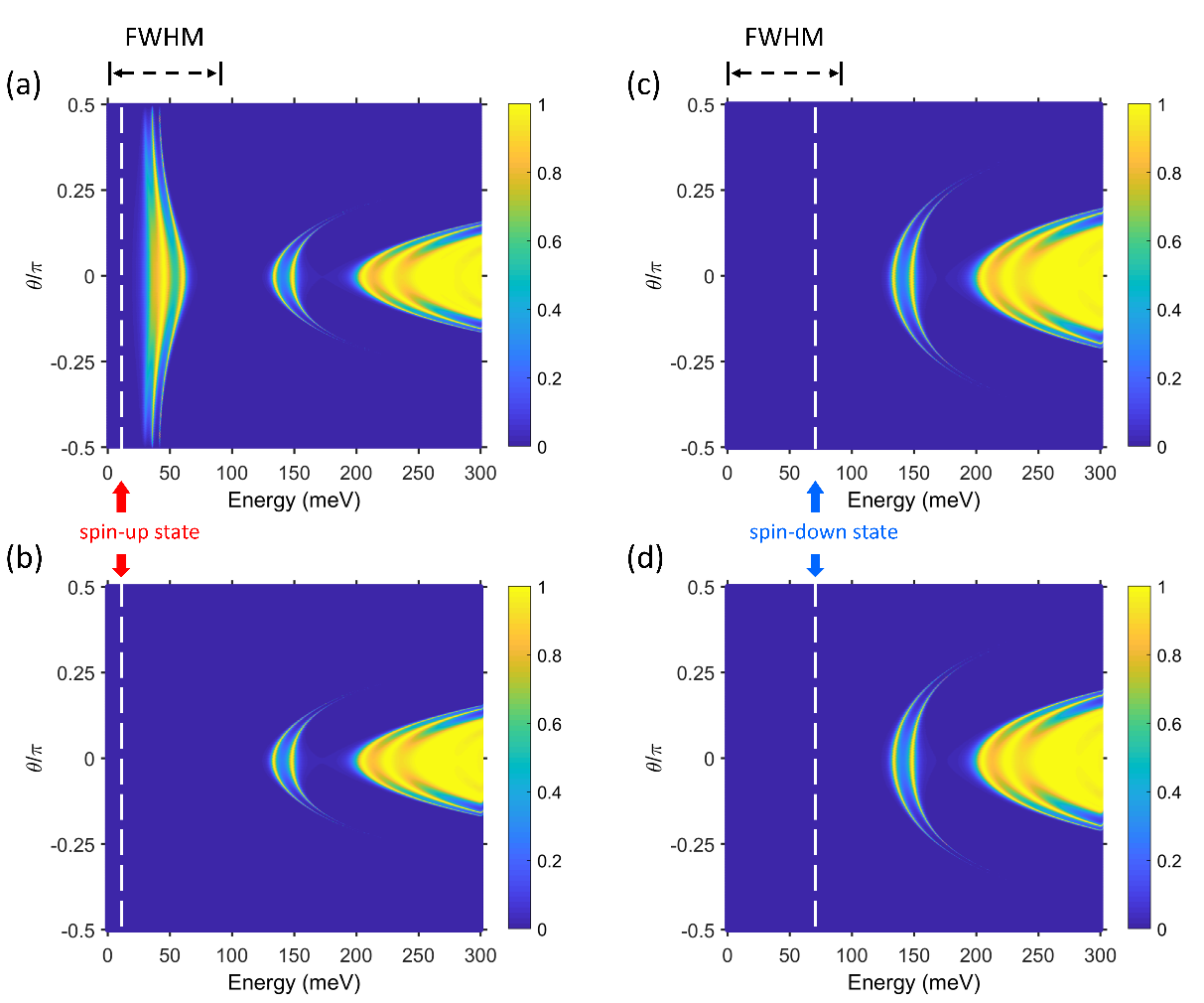


**Fig. S2** Transmission probabilities of each spin state for altering incident energy and incident angle indicated by the green arrow in figure S1b. The transmission probability of the spin-up electrons in the (**a**) parallel and (**b**) antiparallel configurations. (**c**)(**d**) The transmission probability of the spin-down electrons in the parallel and the antiparallel. Here, the length of gate and non-gate region are selected as *w_A_* = 5.4 nm and *w_B_* = 12 nm, respectively. Black dashed lines above the figure are indicate the full width at half-maximum (FWHM) of the Fermi–Dirac function. The white dashed lines and arrows indicate the origination of each spin polarization. The other parameters are the same as those in figure S1.


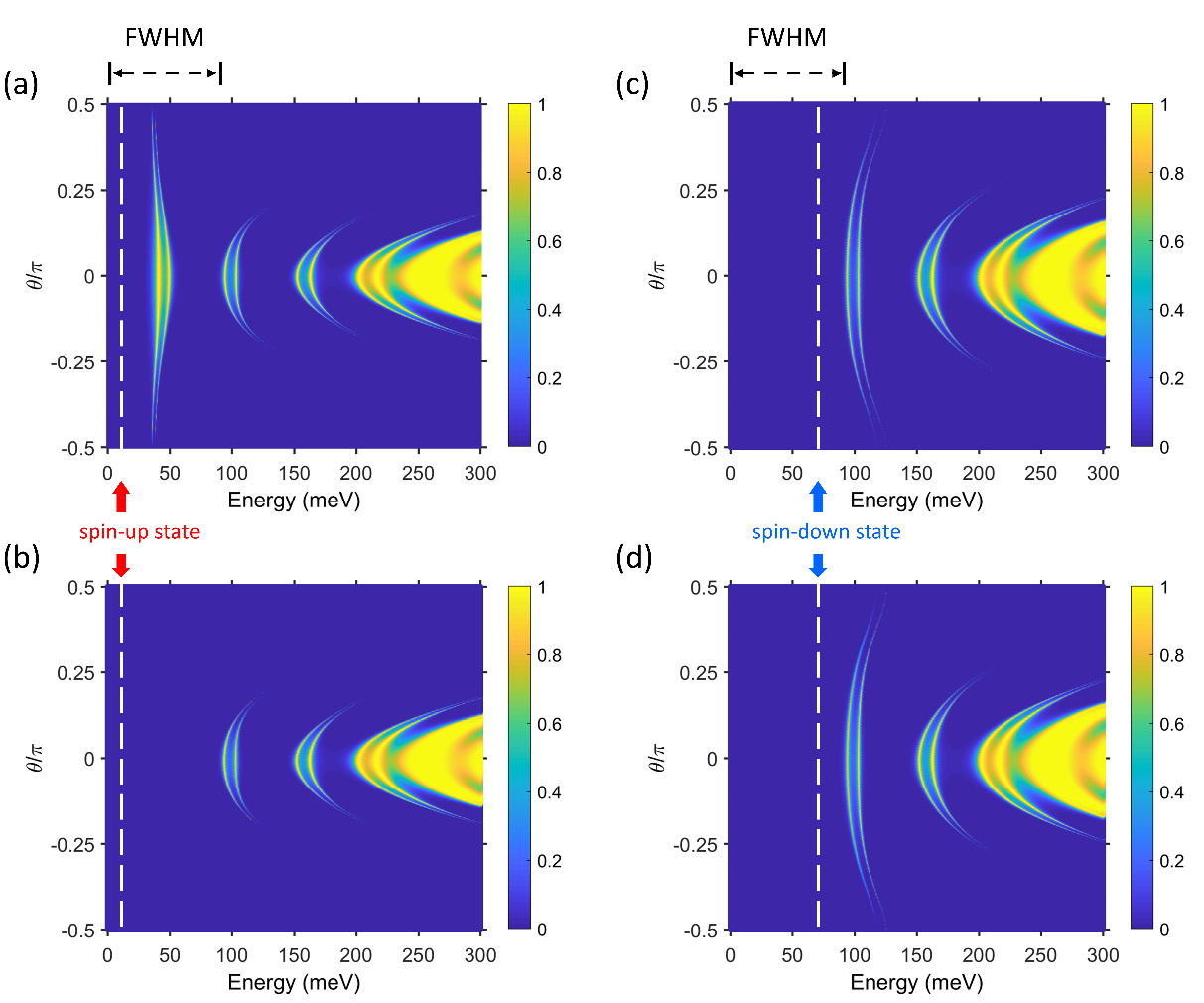


**Fig. S3** Transmission probabilities of each spin state for altering incident energy and incident angle indicated by the brown arrow in figure S1b. The transmission probability of the spin-up electrons in the (**a**) parallel and (**b**) antiparallel configurations. (**c**)(**d**) The transmission probability of the spin-down electrons in the parallel and the antiparallel. Here, the length of gate and non-gate region are selected as *w_A_* = 12 nm and *w_B_* = 12 nm, respectively. The other parameters are the same as those in Fig. S1.


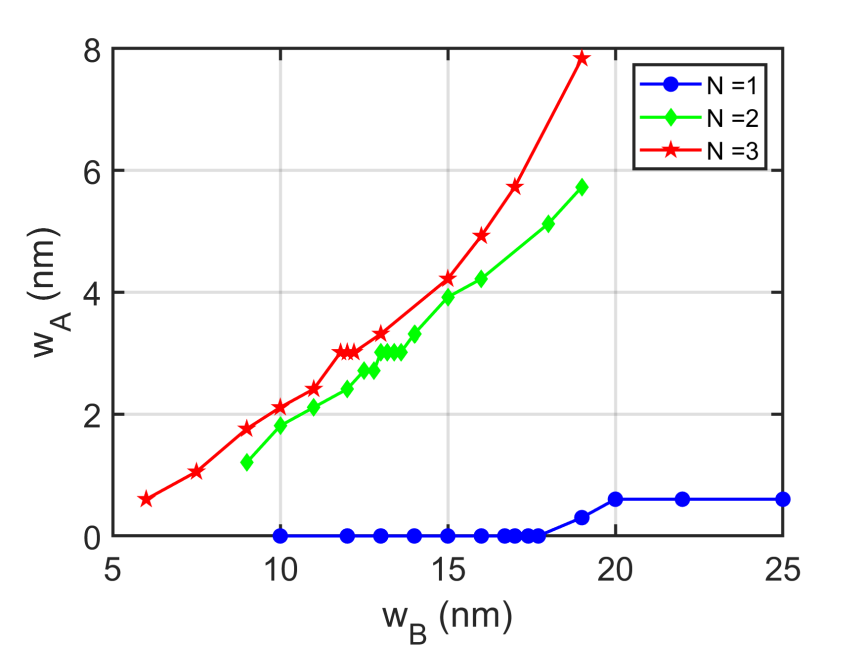


**Fig. S4** Distribution of segment potential of MR. Relationship between gate length *w_A_* and distance between two gates *w_B_* for each MR value in Fig. 3a.


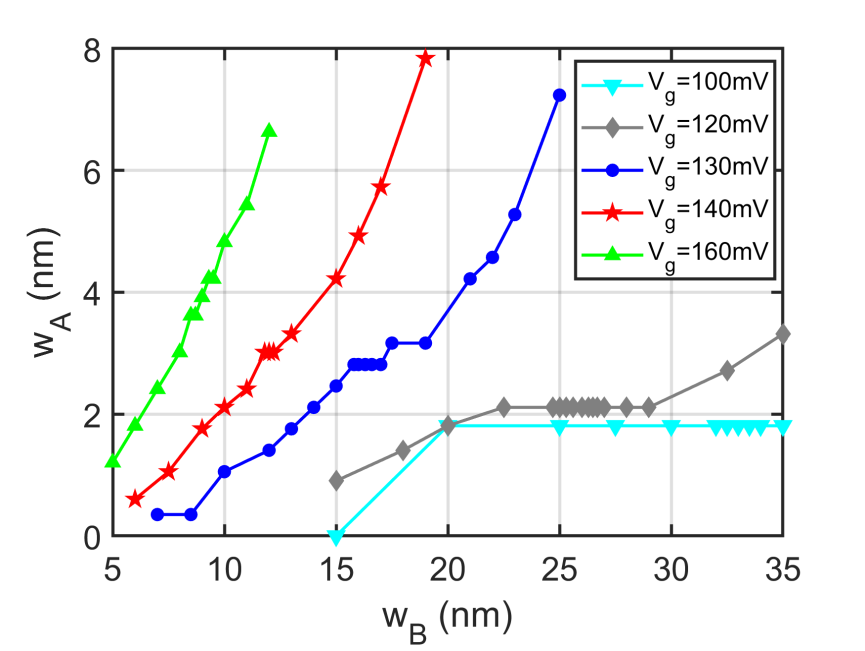


**Fig. S5** Distribution of segment potential of MR. Relationship between gate length *w_A_* and distance between two gates *w_B_* for each MR value in Fig. 3b.
